# Uncovering Illusions of Understanding: A Falsificationist Perspective

**DOI:** 10.1007/s42113-026-00318-3

**Published:** 2026-09-15

**Authors:** Daniel W. Heck

**Affiliations:** Department of Psychology, Philipps-Universität Marburg, Marburg, Germany

## Abstract

In a complex world, scientific explanations are always incomplete. Therefore, can we say that scientists generally suffer from “illusions of understanding?” I argue that embracing a falsificationist perspective makes scientists aware of the provisional nature of all explanations and highlights the limitations of current scientific knowledge. Tools such as preregistration and formal modeling are well suited for uncovering illusions of understanding.

## Explanations are Always Incomplete

Given that the world is infinitely complex, limitations in our understanding of the causes of phenomena are unavoidable (Keil, 2006). A cause is defined by the INUS conditions as “an **I**nsufficient but **N**ecessary part of a condition which is itself **U**nnecessary but **S**ufficient for the result” (Mackie, 1965, p.245). This means that causal explanations themselves are complex and require consideration of multiple causes and boundary conditions. Complete understanding of a phenomenon would require a being with full knowledge of all variables and conditions in a causal system (i.e., Laplace’s demon; Salmon, 1984).

Assuming that multicausality is the norm rather than the exception, science should focus on identifying the most important causes of a phenomenon while treating all negligible causes as “error” (Shiffrin et al., 2026). Such a view implies that many (if not all) explanations are necessarily incomplete (Keil, 2006), analogous to the idea that all models are wrong (Box, 1976). Both explanations and models provide simplified accounts that structure scientific knowledge in order “to deal with real problems” (Box, 1976, p.798).

If all theories and explanations involve simplifications to some degree, this raises the question whether scientists always suffer from “illusions” of understanding. Since all explanations are incomplete and may turn out to be wrong in the future, incomplete and partially wrong explanations should not be framed as “illusions.” Instead, the term “illusion” should be used more narrowly—for instance, to describe scientists who ignore negative empirical evidence or are unaware of the limited scope of their explanations.

## A Falsificationist Perspective

Karl Popper’s (2002) falsificationism provides a natural perspective for thinking about illusions of understanding and for identifying methodological approaches to uncover them. The framework highlights that we can never know whether our explanations are true or false, irrespective of how much supporting evidence we accumulate. Instead, all explanations are provisional, as Shiffrin et al. (2026) also point out: “theories are never ‘correct.’ They are meant to represent what is currently the ‘best’ account” (p. 20). Good theories should have a large scope and make precise predictions about core empirical phenomena deemed to be most interesting and important.

Adopting a falsificationist perspective leads to a more positive and constructive framing of the inherent limitations of scientific knowledge, rather than painting a negative picture in which all scientists fall prey to some kind of illusion. Scientists need to accept and openly communicate that all scientific explanations are provisional and incomplete. Bolstering intellectual humility reduces overconfidence and fosters a critical mindset regarding the limits of our knowledge. Moreover, while scientists often disagree about the *best* explanation, it is much easier to reach consensus about which explanations are *almost certainly wrong*. This asymmetry provides an opportunity to correct at least the most severe illusions of understanding.

## Formal Modeling

The question remains how to learn about the degree to which our provisional explanations are incomplete. For empirical testing, explanations must be specified with sufficient precision to make predictions that may turn out to be wrong (Popper, 2002). Moreover, their assumed scope needs to be clearly delineated to enable fair empirical tests across a broad range of boundary conditions (Bredenkamp, 1980). Formal modeling addresses both goals by specifying theoretical assumptions about psychological processes using mathematical equations. By making precise predictions, formal models allow us to learn whether and where we are wrong (Smaldino, 2017). On the one hand, modeling exposes hidden assumptions, highlights possible misunderstandings about the posited mechanisms, and reveals incorrect derivations of predictions (Harris, 1976). On the other hand, insufficient model fit informs us about the empirical inadequacy and limited scope of our provisional explanations.

Satisfactory model fit is a necessary but not sufficient condition for a model’s validity (Roberts & Pashler, 2000). It is important to critically test the model’s ability to account for new, unseen situations and conditions. Common validation strategies for cognitive models aim exactly in this direction. For instance, tests of selective influence focus on whether theoretically motivated manipulations affect only specific target parameters while leaving the remaining parameters unchanged (e.g., Schmidt et al., 2025). The increased precision of formal models comes with a higher risk of relying on incorrect assumptions (i.e., the Duheme-Quine problem). While a single study cannot disentangle whether core or auxiliary assumptions are incorrect, iterative model validation can shed light on this issue by systematically varying the experimental paradigm.

## Targeted Experimentation and Preregistration

To determine the primary causes and boundary conditions that matter most for a phenomenon, researchers often rely on targeted experimentation. From a falsificationist perspective, it is quintessential to conduct informative empirical tests that critically test the explanations or theories under scrutiny (Bredenkamp, 1980). Informative experimental designs aim to falsify diagnostic predictions rather than to confirm trivial, established regularities (Fiedler, 2017), which helps to uncover—rather than corroborate—illusions of understanding. Given that the search space of possible experimental designs is infinitely large, targeted testing via incremental improvements of experimental paradigms will often be more efficient than random experimentation.

When critically testing provisional explanations, preregistration provides a remedy against cognitive illusions such as hindsight bias or forgetting (Nosek et al., 2018). Preregistration informs the scientific community whether an explanation has survived critical testing or has required post hoc modifications to account for unexpected results. Writing down hypotheses based on our current understanding of a phenomenon also deprives scientists of the opportunity to ignore explanatory failures and facilitates the correction of misunderstandings. Moreover, preregistration requires researchers to think more deeply about the assumed causal mechanisms and the most adequate analysis strategy *before* seeing the data (e.g., whether and how to control for pre-treatment measures). This process may reveal necessary auxiliary assumptions and incomplete chains of reasoning, thus increasing awareness of the “difficulty of deduction” (Shiffrin et al., 2026, p.25). More broadly, by relying on Registered Reports, the soundness of provisional explanations is reviewed by peers, and limitations of our current understanding become transparent and part of the published literature.

## Conclusion

Embracing a falsificationist perspective helps prevent illusions of understanding and increases awareness that all of our knowledge is provisional and incomplete. No single methodological tool—neither formal modeling nor preregistration—can guarantee that we arrive at deep and sufficiently adequate explanations of the causes of a phenomenon. Still, these tools are beneficial for uncovering the most severe illusions of understanding.

## Data Availability

No datasets were generated or analysed during the current study.

## References

[CR1] Box, G. E. P. (1976). Science and statistics. *Journal of the American Statistical Association,**71*, 791–799. 10.1080/01621459.1976.10480949

[CR2] Bredenkamp, J. (1980). *Theorie und Planung psychologischer Experimente*. Darmstadt: Steinkopff.

[CR3] Fiedler, K. (2017). What constitutes strong psychological science? The (neglected) role of diagnosticity and a priori theorizing. *Perspectives on Psychological Science,**12*, 46–61. 10.1177/1745691616654458 28073328

[CR4] Harris, R. J. (1976). The uncertain connection between verbal theories and research hypotheses in social psychology. *Journal of Experimental Social Psychology,**12*, 210–219. 10.1016/0022-1031(76)90071-8

[CR5] Keil, F. C. (2006). Explanation and understanding. *Annual Review of Psychology,**57*, 227–254. 10.1146/annurev.psych.57.102904.190100 16318595 PMC3034737

[CR6] Mackie, J. L. (1965). Causes and conditions. *American Philosophical Quarterly*, *2*, 245–264. https://www.jstor.org/stable/20009173

[CR7] Nosek, B. A., Ebersole, C. R., DeHaven, A. C., & Mellor, D. T. (2018). The preregistration revolution. *Proceedings of the National Academy of Sciences,**115*, 2600–2606. 10.1073/pnas.1708274114 PMC5856500 29531091

[CR8] Popper, K. (2002). *The logic of scientific discovery*. Routledge. 10.4324/9780203994627

[CR9] Roberts, S., & Pashler, H. (2000). How persuasive is a good fit? A comment on theory testing. *Psychological Review,**107*, 358–367. 10.1037/0033-295X.107.2.358 10789200

[CR10] Salmon, W. C. (1984). *Scientific explanation and the causal structure of the world*. Princeton University Press. 10.2307/j.ctv173f2gh

[CR11] Schmidt, O., Erdfelder, E., & Heck, D. W. (2025). How to develop, test, and extend multinomial processing tree models: A tutorial. *Psychological Methods,**30*, 720–743. 10.1037/met0000561 37498691

[CR12] Shiffrin, R. M., Stigler, S., & Keil, F. (2026). Illusions of understanding in the sciences. *Computational Brain & Behavior.* 10.1007/s42113-026-00271-1

[CR13] Smaldino, P. E. (2017). Models are stupid, and we need more of them. In: R. R. Vallacher et al (eds) *Computational Social Psychology*. Taylor and Francis, pp 311–331.

